# Interatrial Septal Mass Secondary to Rosai-Dorfman Disease: A Rare Cause of Complete Heart Block

**DOI:** 10.1016/j.jaccas.2023.101824

**Published:** 2023-04-21

**Authors:** Abdul Hadi Butt, Mark Peterzan, Chirag Shah, Matthew Wright, Christopher A. Rinaldi, Jaswinder Gill, Stamatis Kapetanakis, Jonathan M. Behar

**Affiliations:** Guy’s and St Thomas’ National Health Service Foundation Trust, London, England

**Keywords:** imaging, magnetic resonance sequences, ultrasound, cardiac pacemaker

## Abstract

Varying degrees of atrioventricular block can be associated with old age or a manifestation of an ischemic, metabolic, or infective pathology. In patients with no clear explanation, it is important to investigate secondary causes. Our case describes the first case of an adult with Rosai-Dorfman histiocytosis presenting with complete heart block. (**Level of Difficulty: Advanced.**)

## Graphical abstract

**Figure:**
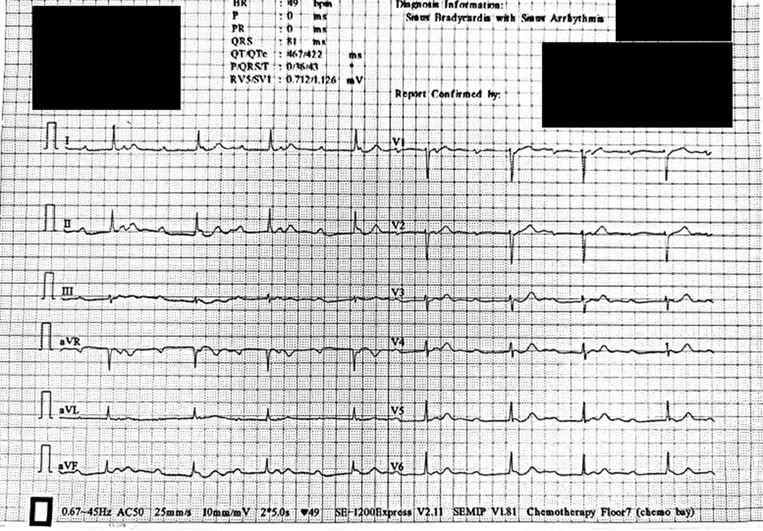

## History of Presentation

**Figure 1:**
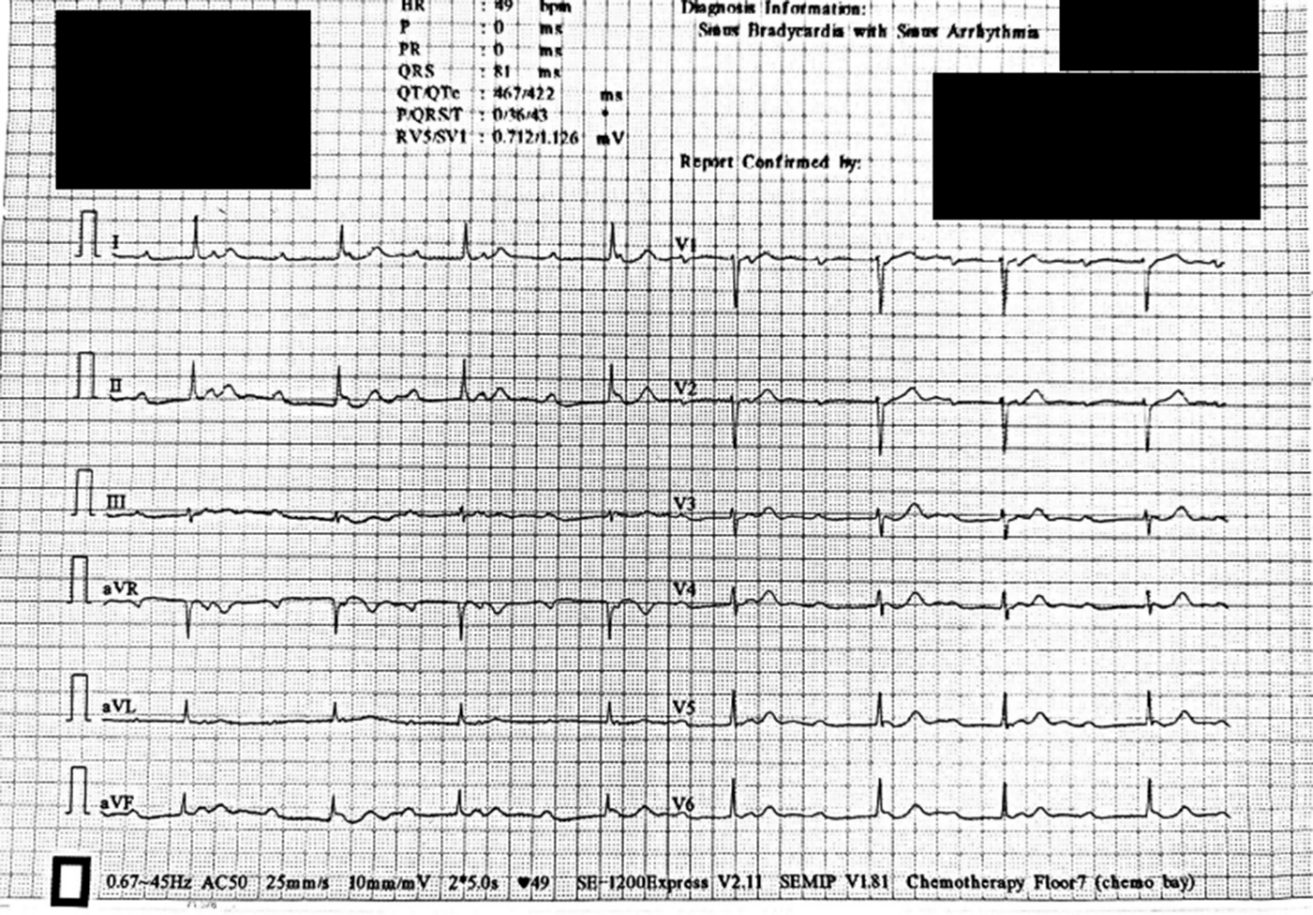
Admission ECG Initial ECG demonstrating complete AV dissociation. AV = atrioventricular; ECG = electrocardiogram.

A 54-year-old, African woman presented with a 2-month history of chest tightness with associated shortness of breath and dizziness. An electrocardiogram was conducted that showed third-degree heart block, prompting further investigation (Figure 1).

**Learning Objectives**

• To describe the presentation of a rare hematologic disorder presenting with an IAS mass.
• To demonstrate the utility of multimodality imaging for identifying the etiology of an intracardiac mass.

## Medical History

She had a background of Rosai-Dorfman disease (RDD) with bony involvement, type 2 diabetes mellitus, and first-degree atrioventricular (AV) block. Her only medication history was metformin 500 mg twice a day. Previously, she had a histiocytic lesion in the right hip that resulted in a fragility fracture requiring a right total hip replacement. Furthermore, there was a lesion in the left hip that was being monitored and a lesion in the left sacrum that required radiotherapy.

## Differential Diagnosis

Differential diagnosis included all causes of pathologic blockade of AV conduction, including infective, metabolic, pharmacologic, and structural disturbances.

## Investigations

Initial blood results demonstrated a normal blood count, electrolytes, and thyroid function, and chest x-ray appeared normal with no signs of infection. Due to a high index of suspicion for structural cardiac disease, it was decided to carry out cardiac magnetic resonance imaging (MRI) in the first instance.

Cardiac MRI was performed using a 1.5-T Siemens Aera scanner; steady-state free precession cine imaging, T1-weighted imaging pre- and post-gadolinium-based contrast, T2-weighted imaging, pre-contrast T1 and T2 mapping, and early- and late-gadolinium enhancement images were acquired.

**Figure 2:**
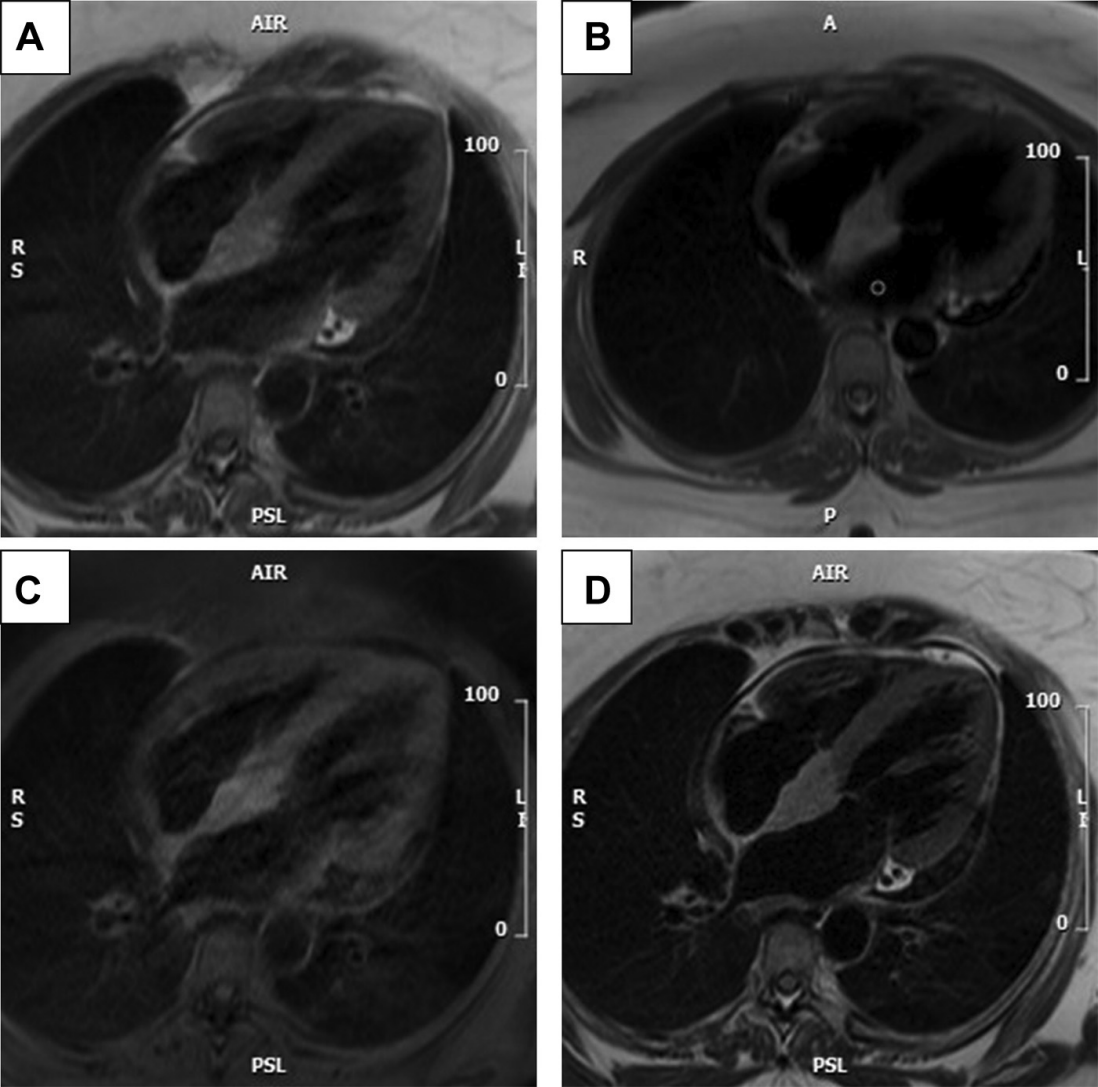
Noncontrast MRI Sequences **(A)** Half-Fourier Acquisition Single-shot Turbo spin Echo imaging showing a relative high signal in the IAS compared with myocardium; **(B)** T1 weighting showing hyperintensity in the IAS compared with myocardium; **(C and D)** T1 and T2 with fat suppression showing persistent high signal in the IAS. IAS = interatrial septum; MRI = magnetic resonance imaging.

The interatrial septum (IAS) was thickened by an irregular mass (37 × 23 mm) encroaching onto the basal interventricular septum and extending from the level of the coronary sinus up to the aortic valve annulus (Figure 2A and 2B). The mass was high-signal on Half-Fourier Acquisition Single-shot Turbo spin Echo imaging, isointense compared with myocardium on balanced–steady-state free precession imaging, hyperintense compared with myocardium on T1-weighted imaging (Figure 2C) and T2-weighted imaging (Figure 2D), and no different after spectral presaturation of fat signal. On precontrast parametric mapping, the mass had high T1 (∼1251 ms) and T2 (∼80 ms) values. The mass showed heterogenous enhancement in the early and late phases after gadolinium contrast

**Figure 3:**
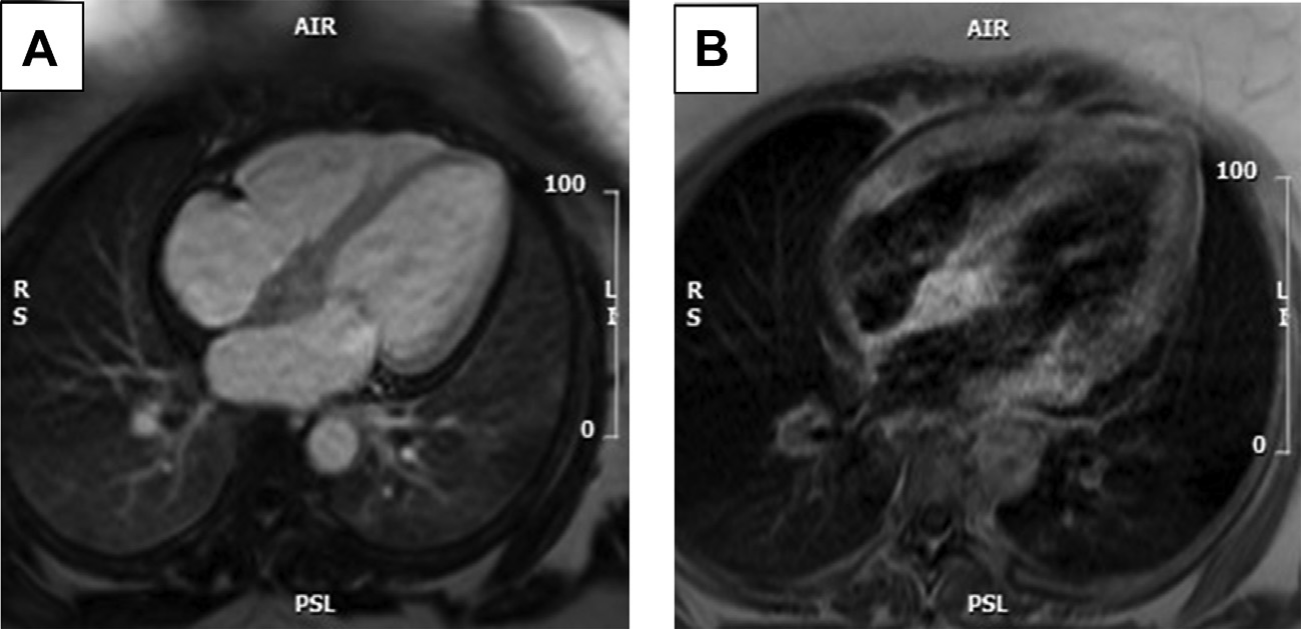
MRI With Gadolinium **(A and B)** showing low early gadolinium enhancement of the IAS and high signal late. Abbreviations as in Figure 2.

Biventricular indexed end-diastolic volume and systolic function were normal (left ventricular ejection fraction: 54%; right ventricular ejection fraction: 57%). Outside of the mass, there was no evidence of myocardial edema on T2w imaging, no intracardiac thrombus on long TI early gadolinium enhanced imaging, and no myocardial infarction or fibrosis on late gadolinium imaging (Figure 3). Biatrial size was normal. There was mild aortic and mitral regurgitation. Incidental low volume axillary and supraclavicular lymphadenopathy were noted (Figure 3).

The impression was that the intracardiac mass represented an extranodal manifestation of the known non-Langerhans histiocytosis and had imaging characteristics that would be consistent with this. Its location was also consistent with the presentation of complete heart block.

**Figure 4:**
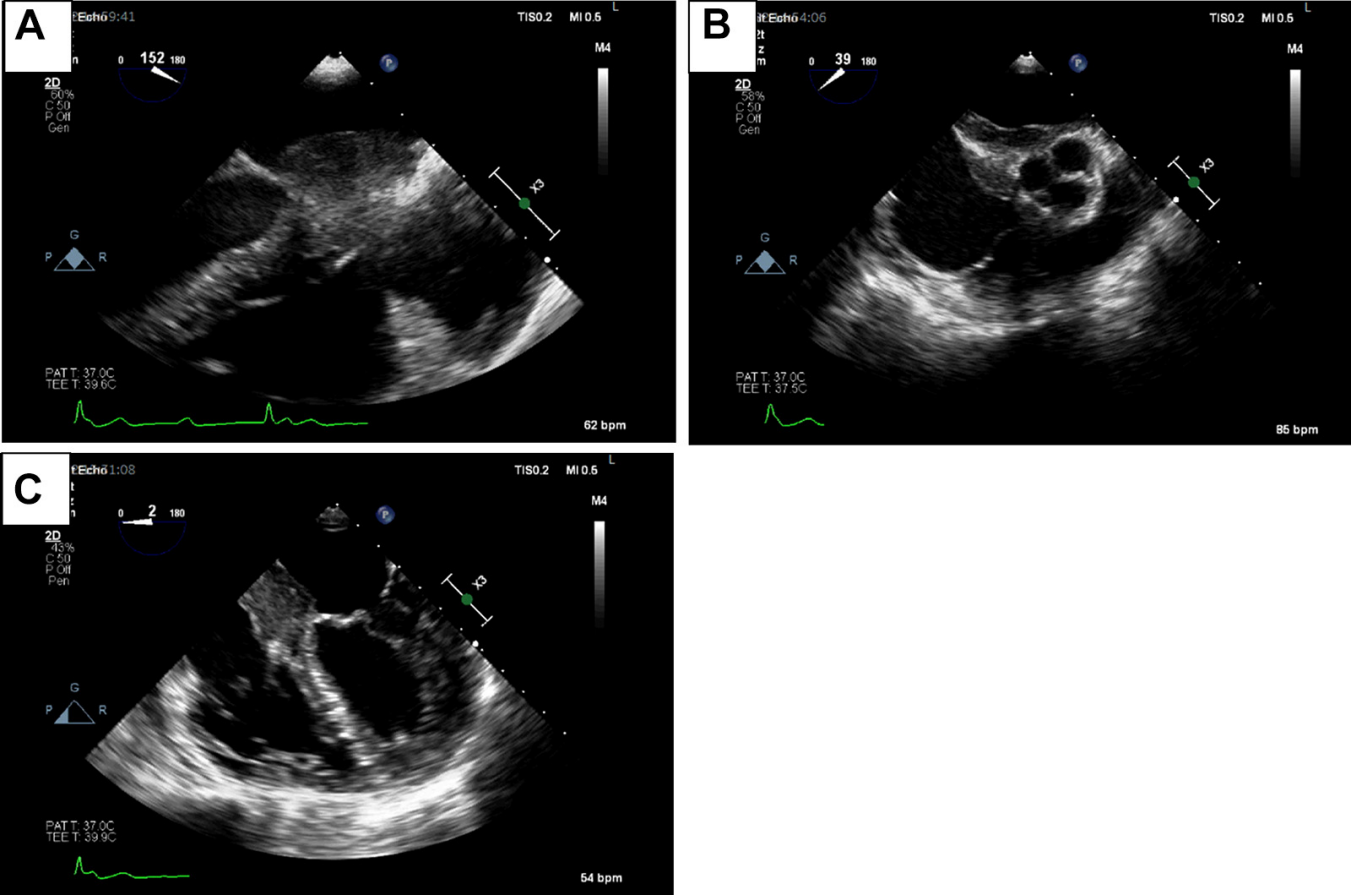
Transesophageal Echocardiogram Transesophageal echo demonstrating the extent of the IAS mass. Abbreviation as in Figure 2.

Following detailed tissue characteristics from the MRI, a transesophageal echocardiogram was carried out to detail the structure of the intracardiac mass. The transesophageal echocardiogram corroborated the findings from the MRI of an infiltrative IAS mass (Figure 4). The mass effect was, therefore, established as the cause of the AV dissociation (Figure 4).

**Figure 5:**
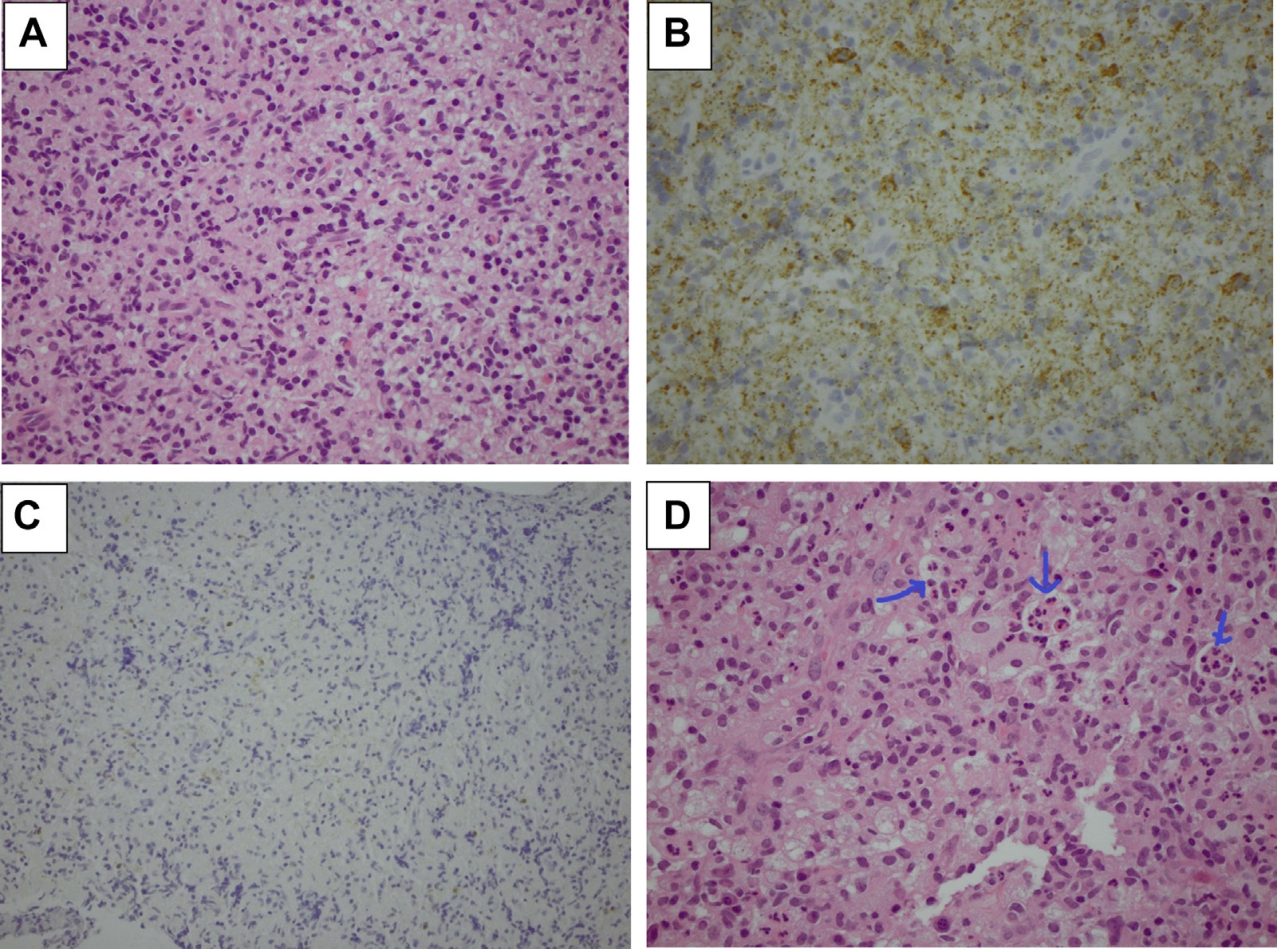
Histology From Biopsied Lesions **(A)** Hematoxylin and eosin stain of endomyocardial biopsy specimen showing sheets of histiocytes; **(B)** PGM staining showing sheets of macrophages; **(C)** S100 stain negative; and **(D)** left iliac biopsy taken prior to this presentation showing emperipolesis on hematoxylin and eosin stain. PGM1 = phosphoglucomutase 1.

To further investigate the IAS mass, an endomyocardial biopsy was carried out. The myocardial biopsy specimens of the IAS mass showed diffuse infiltration by large numbers of histiocytes with an abundance of foamy eosinophilic cytoplasm on hematoxylin and eosin staining (Figure 5A).

Also noted were many small mature lymphocytes, few plasma cells, and occasional eosinophils and neutrophils. No granulomas were seen, and no obvious emperipolesis was identified. No micro-organisms were seen with special stains (Gram, Grocott, Ziehl-Nielsen, and Periodic acid–Schiff–diastase stains). Immunostaining phosphoglucomutase 1 highlighted abundant histiocytes (Figure 5B and 5C). S100, Cyclin D1, and CD1a were negative. There was no increase in immunoglobulin G4 positive cells seen.

Although the classically seen emperipolesis was not present on this biopsy, our patient did have a previous left iliac biopsy showing emperipolesis (Figure 5D). As such, in view of the endomyocardial biopsy demonstrating a large number of histiocytes together with the clinical presentation, it was deemed that the patient had developed complete heart block secondary to a new invasive mass secondary to her Rosai-Dorfman histiocytosis.

## Management

Given the location of the IAS mass, it was agreed that resection would not be feasible due to its association with surrounding structures including mitral apparatus, interventricular septum, and coronary sinus. Medical management of the RDD was discussed but, because the progression of the disease was uncertain, it was agreed that on balance the patient should be offered a dual-chamber pacemaker. Ten months later she presented with clinical heart failure and significantly impaired left ventricular systolic function most likely as a result of pacing-induced cardiomyopathy. She underwent an attempted upgrade to biventricular pacing, however, we were unable to successfully cannulate the coronary sinus and she was implanted with a left bundle area pacing lead for resynchronization.

## Discussion

RDD is an uncommon histiocytic disorder, which classically presents with massive bilateral painless cervical lymphadenopathy associated with weight loss, fevers, and night sweats.^1^ Most seen in African patients with a male predominance, the disease can be split into either sporadic or familial and cutaneous RDD.^1^

Extranodal disease has been reported in >40% of patients with common sites being the skin, nasal cavity, bone, orbital tissue, and central nervous system.^1^ Even more rarely, the disease can present with cardiac involvement, in a reported 0.1%-0.2% of cases.^2^ This usually manifests as an intracardiac mass and, in the cases reported, often represents multifocal underlying cardiac involvement.^2^ From an electrophysiological standpoint, there is report of 2 children with RDD manifesting with complete heart block^3^^,^^4^ and 1 adult presenting with an electrical storm.^5^

Given the scarcity of reported cardiac involvement of RDD, the optimal approach to management remains unclear. The most recent consensus of management (2018) suggests that the disease progression is often unpredictable, and that extranodal involvement is often associated with a poor prognosis.^6^

In our case, the presence of first-degree heart block 2 years before any cardiac imaging suggests there had been intracardiac involvement of Rosai-Dorfman that was not rapidly expanding. This was in the context of no systemic treatment for the disease because the previously discovered bony lesion was managed with localized radiotherapy. This may give some indication of the natural progression of the disease, but further studies are required before this can be confirmed.

## Follow-up

After implantation of the permanent pacemaker, pacing parameters remained stable, however, she developed clinical heart failure with new depression of left ventricular systolic function hence the decision for upgrade to resynchronization. Our patient was offered systemic steroid treatment as an attempt to limit the growth of the interatrial mass, but this was declined due to the side effect profile of systemic glucocorticoids. As such, the patient will remain under follow-up and is planned for interval imaging to assess the progression of the mass.

## Conclusions

Understanding the pathophysiology remains a key component to managing patients with complete heart block and this is highlighted in the current European Society of Cardiology 2021 pacing guidelines.^7^ This case demonstrates the value of multimodality cardiac imaging in a young patient with RDD to allow for identification of cardiac involvement. Management of masses should be agreed using input from multiple specialists given the lack of data to guide treatment.

## Funding Support and Author Disclosures

The authors have reported that they have no relationships relevant to the contents of this paper to disclose.

## Appendix

Video 1 Transesophageal Echo 4-Chamber View Four-chamber view demonstrating extent of IAS mass.
